# Enucleation and Evisceration: A 10-Year Analysis of Postoperative Complications and Sympathetic Ophthalmia Risk at a Major Australian Tertiary Hospital, With a Review of the Current Literature

**DOI:** 10.7759/cureus.82646

**Published:** 2025-04-20

**Authors:** Vivien Nguyen, Alexandra I Manta, Phung Vu

**Affiliations:** 1 Ophthalmology, Princess Alexandra Hospital, Brisbane, AUS

**Keywords:** enucleation, evisceration, orbital implant, postoperative complications, sympathetic ophthalmia

## Abstract

Purpose: This study aims to compare postoperative outcomes between enucleation and evisceration surgery over a 10-year period at an Australian tertiary public hospital.

Methods: Retrospective chart review of patients who underwent primary enucleation or evisceration surgery at the Princess Alexandra Hospital between 1st March 2014 and 1st March 2024. After inclusion and exclusion criteria were applied, 73 eyes remained in the study (45 evisceration; 28 enucleation).

Results: Larger-sized implants were used in the enucleation cohort compared to the evisceration cohort (p=0.011). Enucleation surgeries (35.7%) had more postoperative complications than the evisceration group (15.6%) (p=0.004). The enucleation cohort had a higher prevalence of implant complications (exposure or extrusion) (21.7%) compared to the evisceration cohort (3.1%) (p=0.029). The prevalence of implant complications between evisceration and enucleation was not statistically significant for birth sex, race, surgery indication, implant material, or implant size. There was no reported sympathetic ophthalmia (SO) in either cohort.

Conclusions: There was no reported SO among the two groups. Patients who underwent enucleation surgery were, however, significantly more at risk of experiencing a postoperative complication and implant exposure or extrusion. There is poor follow-up compliance in patients who have undergone anophthalmic surgery in the Australian public hospital system. Improving patient education and surgical guidelines may help achieve better postoperative outcomes and follow-up durations after anophthalmic surgery.

## Introduction

The debate over when to opt for enucleation versus evisceration continues to persist in the oculoplastic community [1-5]. Enucleation involves the removal of the entire globe, whereas evisceration is the removal of only the intraocular contents while leaving the sclera, conjunctiva, extraocular muscles, and remaining orbital tissues behind [1,4,6,7]. Both procedures offer distinct advantages and considerations, with the decision largely dependent on the clinical context, patient-specific factors, and surgeon preference. These surgeries may be indicated in painful, blind, or phthisical eyes; however, enucleation is preferred in intraocular malignancy or trauma cases with significant tissue loss and uveal exposure where there is risk of sympathetic ophthalmia (SO) [2]. Literature has compared the outcomes of enucleation and evisceration surgeries, often focusing on factors such as postoperative complications, cosmesis, and patient satisfaction [6,7]. Evisceration is known to have better cosmesis, improved implant motility, and fewer postoperative complications but is thought to hold a higher risk of SO development than enucleation [6]. Postoperative outcomes appear to vary depending on geographical location and cultural environment, as these factors can affect the indication for surgery and/or surgeon preference towards a certain operation [1,2,4]. While there has been literature comparing demographic data and clinical outcomes between enucleation and evisceration surgeries in both developing and developed countries, there is no longitudinal data within an Australian landscape. Previous studies have compared surgical indications and implant types on the rate of postoperative complications, such as implant extrusion; however, they were limited by small sample sizes and had not investigated the impact of implant size [8]. Studies that had larger sample sizes and greater longitudinal data only focused on general demographic and surgery characteristics and did not analyze how these potential variables may affect postoperative outcomes and complications. There have been reports analyzing implant exposure and extrusion rates post-anophthalmic surgery; however, this was limited to patients who had undergone surgery for endophthalmitis only or had investigated standalone evisceration or enucleation surgeries without comparing the two groups [9,10]. This study will therefore aim to provide more clinical context on the postoperative outcomes, such as postoperative and implant complication rates, between patient groups who have undergone enucleation or evisceration surgery over 10 years at a major Australian tertiary public hospital. Specifically, this study will look at the potential effect that demographics (e.g., age, sex, race), surgery indication, implant size, and implant material may have on postoperative complications, including SO and implant exposure. The results from this study will aim to provide clinically relevant information to assist in anophthalmic surgery decision-making and the development of guidelines to improve postoperative outcomes.

## Materials and methods

Study design, population, and settings

Retrospective data were obtained from patients who underwent enucleation or evisceration surgery at the Princess Alexandra Hospital, a major tertiary Australian public hospital, between 1st March 2014 and 1st March 2024. This data was obtained using surgical procedural coding for primary enucleation or evisceration surgery collected by the Princess Alexandra Hospital medical records department. Inclusion criteria included adults aged > 18 years who had either primary enucleation or evisceration surgery between 1st March 2014 and 1st March 2024. Participants who were <18 years old, had no documented preoperative visual acuity (VA), had no documented reason for surgery, or had prior anophthalmic surgery were excluded. After inclusion and exclusion criteria were applied, there were 73 participants (73 eyes) remaining in the study. The demographic, clinical characteristics, and ophthalmic examination findings were collected via the integrated electronic medical record (iEMR). Ethics approval for the study was granted by the Institutional Human Research Ethics Committee (HREC/2024/QMS/107381). This study adhered to the principles outlined in the Declaration of Helsinki. All aspects of the study were kept confidential, and only researchers in this study had authorized access.

Study measures

Demographic, Operative Details, and Postoperative Outcomes

Demographic information, age at time of surgery, birth sex, and racial background were obtained from the iEMR. Racial background was classified into two groups: Caucasian individuals and Aboriginal and Torres Strait Islander or Other (Asian, African, Pacific Islander) individuals. Clinical characteristics collected included preoperative VA, surgery indication, and days to surgery, which was calculated as the days between the decision for surgery and the operation date. Data on the principal surgeon (senior ophthalmologist vs. trainee), urgency of surgery (emergency vs. elective), use of an orbital implant, implant material, and implant size were also collected. Postoperative details, including one-year follow-up outcome, postoperative complications, and any subsequent implant revisions, were obtained from iEMR. These details were based on ophthalmic examination by the clinician who saw the patient at the given time of review. Postoperative complications included implant exposure, implant extrusion, inflammation or infection requiring medical intervention, hematoma, ptosis, or wound dehiscence. Wound dehiscence was defined as not requiring surgical intervention and having no evidence of obvious implant exposure or extrusion. The medical charts were reviewed up until one year post-surgery unless a follow-up outcome had been determined prior to this timeframe (e.g., discharged, lost to follow-up, referred to ocularist, seen in private practice, ongoing review). This time frame was chosen to allow comparison of postoperative outcomes within a standardized period, as many patients did not have follow-up past the one-year postoperative mark.

Data analyses

The collected data were analyzed using IBM SPSS Statistics for Windows, Version 27 (Released 2020; IBM Corp., Armonk, New York, United States). Descriptive statistics, including means, standard deviations, frequencies, and percentages, were calculated to summarize population demographics and clinical characteristics. Inferential statistical analyses were conducted to evaluate associations and differences between evisceration and enucleation groups. Categorical variables were compared using the chi-square test. Continuous variables were assessed using the independent samples t-test. A two-sided p-value of <0.05 was considered statistically significant. Subgroup analyses were conducted to compare differences between the two surgical procedures across demographic variables (e.g., age, birth sex, race), operative factors (e.g., presence of an orbital implant, implant material, implant size), and postoperative complications. 

## Results

Participants’ demographic and clinical characteristics

A total of 73 eyes were included in the study: 45 in the evisceration cohort and 28 in the enucleation cohort. There was no significant difference in mean age, birth sex, or preoperative VA between the two groups. The mean age at surgery was similar between the groups: 70.7 years old in the evisceration cohort and 64.1 years old in the enucleation cohort. The majority of patients who had evisceration were female (n=25, 55.6%), while the majority who had enucleation were male (n=15, 53.6%), although this difference was not clinically or statistically significant. Both cohorts had a pre-operative VA worse than hand movements. There was a statistical difference in the racial breakdown: 97.8% (n=44) were Caucasian individuals and 2.2% (n=1) were Aboriginal and Torres Strait Islander or Other individuals in the evisceration cohort, while 78.6% (n=22) were Caucasian individuals and 21.4% (n=6) were Aboriginal and Torres Strait Islander or Other individuals in the enucleation cohort (p=0.007). Table 1 provides a summary of the demographic data and indications for surgery. The main indications for surgery in the evisceration cohort were non-healing corneal perforation (n=21, 46.7%) and painful blind eye (n=17, 37.8%), whereas in the enucleation cohort, it was painful blind eye (n=11, 39.3%) and trauma (n=7, 25.0%) (p=0.022).

**Table 1:** Demographic and clinical characteristics of evisceration and enucleation cohorts. VA: visual acuity; >HM: worse than hand movements; <HM: better than or including hand movements

Category	Evisceration (n=45)	Enucleation (n=28)	p-value
Mean Age (years)	70.7	64.1	0.081
Sex			0.448
Female	25 (55.6%)	13 (46.4%)	
Male	20 (44.4%)	15 (53.6%)	
Race			0.007
Caucasian	44 (97.8%)	22 (78.6%)	
Aboriginal/Torres Strait Islander or Other	1 (2.2%)	6 (21.4%)	
Pre-op VA			0.458
>HM	37 (82.2%)	21 (75.0%)	
≤HM	8 (17.8%)	7 (25.0%)	
Surgery Indication			0.022
Non-healing corneal perforation	21 (46.7%)	4 (14.3%)	
Malignancy	0 (0.0%)	4 (14.3%)	
Painful blind eye	17 (37.8%)	11 (39.3%)	
Phthisical eye	3 (6.6%)	2 (7.1%)	
Trauma	4 (8.9%)	7 (25.0%)	

Operative details between evisceration and enucleation

The mean days to surgery were similar between evisceration (29.5 days; range 0-388 days) and enucleation (41.4 days; 0-503 days) (p=0.583). The majority of evisceration was conducted as emergency cases, whereas the majority of enucleation was done as an elective procedure (p=0.023). An orbital implant was used in the majority of cases, with an acrylic implant being the most common material amongst both cohorts. There was a statistical difference in implant sizes used between the evisceration and enucleation cohorts. The 20mm (n=21, 65.5%) and 18mm (n=5, 15.6%) sized implants were the majority in patients who underwent evisceration, while the majority used in the enucleation group were the 22mm (n=12, 52.2%) and 20mm (n=8, 34.8%) sized implants (p=0.011) (Table 2).

**Table 2:** Operative details (surgeon experience, use of an orbital implant, implant material, implant size) of evisceration and enucleation cohorts.

Operative details		Evisceration (n=45)	Enucleation (n=28)	p-value
Urgency (n=73)	Emergency	29 (64.4%)	9 (32.1%)	0.023
	Elective	16 (35.6%)	19 (67.9%)	
Principal surgeon (n=73)	Senior ophthalmologist	13 (28.9%)	8 (28.6%)	0.890
	Trainee	32 (71.1%)	20 (71.4%)	
Implant (n=73)	Yes	32 (71.1%)	23 (82.1%)	0.288
	No	13 (28.9%)	5 (17.9%)	
Implant material (n=55; evisceration=32, enucleation=23)	Acrylic	28 (87.5%)	21 (91.3%)	0.633
	Glass	1 (3.1%)	0 (0.0%)	
	Porous polyethylene	3 (9.4%)	2 (8.7%)	
Implant size (n=55; evisceration=32, enucleation=23)	16mm	3 (9.4%)	1 (4.3%)	0.011
	18mm	5 (15.6%)	2 (8.7%)	
	20mm	21 (65.5%)	8 (34.8%)	
	22mm	3 (9.4%)	12 (52.2%)	

Postoperative details between evisceration and enucleation

There was no statistical difference in the one-year follow-up outcomes between the evisceration and enucleation cohorts, with most patients lost to follow-up (evisceration 48.9%; enucleation 25.0%) or having been referred onwards to an ocularist (evisceration 20.0%; enucleation 35.7%). In total, seventeen patients had experienced a postoperative complication, with enucleation (n=10, 35.7%) having more postoperative complications than evisceration (n=7, 15.6%) (p=0.004). The mean duration to a postoperative complication was, however, similar between the evisceration (3.9 months; range 0 - 11.5 months) and enucleation (2.6 months; range 0 - 15.9 months) cohorts and was not statistically significant. Six patients experienced implant complications: five enucleation patients had implant exposure, while one evisceration patient had implant extrusion. Patients who had undergone enucleation had a significantly higher prevalence of implant complications (n=5, 21.7%) compared to those who had undergone evisceration (n=1, 3.1%) (p=0.029). Out of the five patients who had enucleation and experienced implant complications, three did not have implant revision surgery for the following reasons: one was lost to follow-up, one had declined revision, and one was on the surgical waitlist at the time of data collection (Table 3).

**Table 3:** Postoperative details (one-year follow-up outcome, postoperative complication, implant exposure/extrusion, implant revision) of enculeation and evisceration cohorts.

Postoperative details		Evisceration (n=45)	Enucleation (n=28)	p-value
1-year follow-up outcome (n=73)	Discharged	2 (4.5%)	5 (17.9%)	0.094
	Lost to follow-up	22 (48.9%)	7 (25.0%)	
	Ocularist follow-up	9 (20.0%)	10 (35.7%)	
	Ongoing review	6 (13.3%)	3 (10.7%)	
	Private practice	6 (13.3%)	3 (10.7%)	
Postoperative complication (n=73)	Yes	7 (15.6%)	10 (35.7%)	0.004
	No	38 (84.4%)	18 (64.3%)	
Months to postoperative complication (n=17)		3.9	2.6	0.490
Implant exposure or extrusion (n=55, evisceration=32, enucleation=23)	Yes	1 (3.1%)	5 (21.7%)	0.029
	No	31 (96.9%)	18 (78.3%)	
Implant revision (n=55, evisceration=32, enucleation=23)	Yes	1 (3.1%)	2 (8.7%)	0.565
	No	31 (96.9%)	21 (91.3%)	

Postoperative complications between evisceration and enucleation

**Figure 1:**
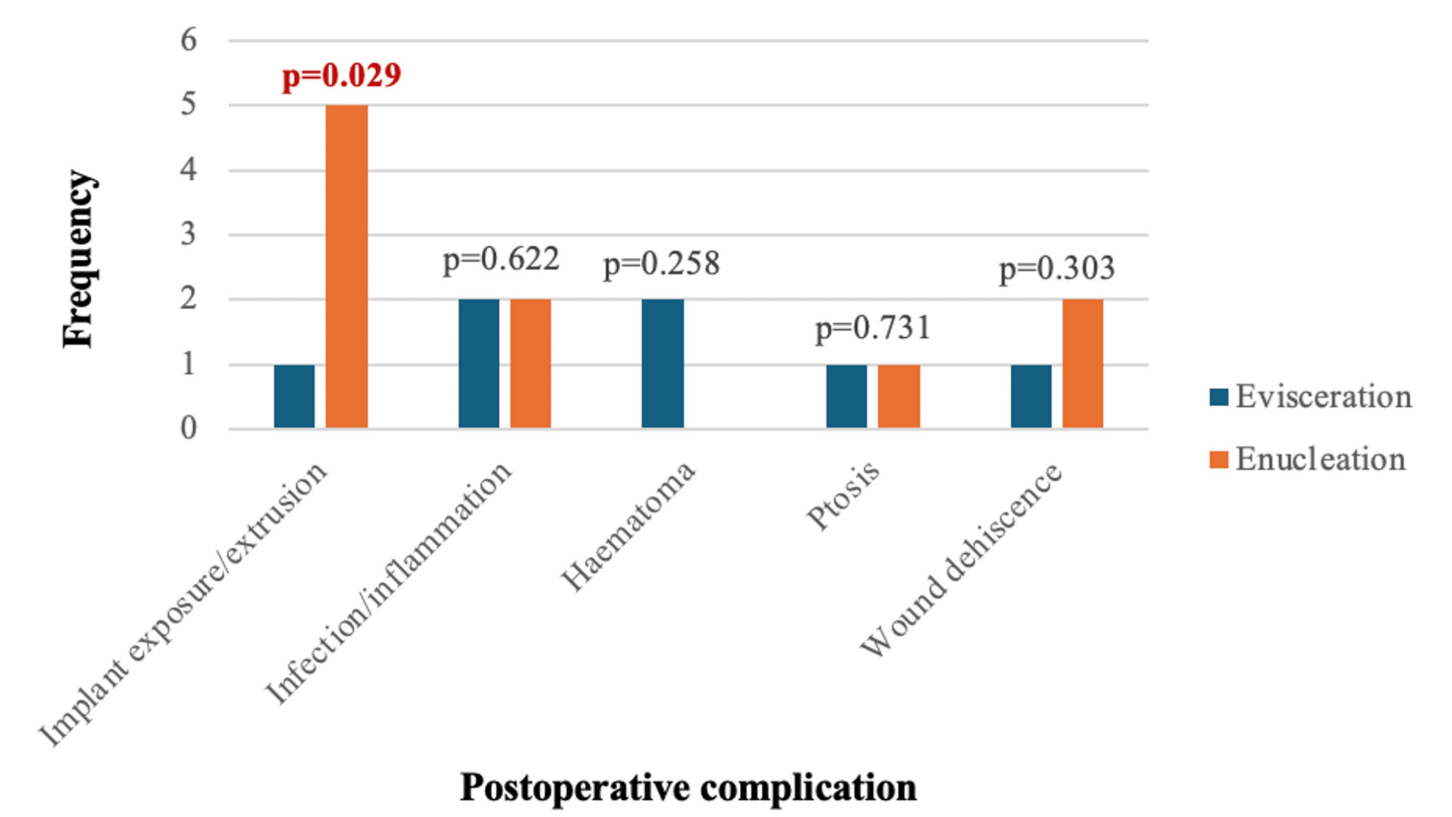
The frequency of postoperative complications (implant exposure/extrusion, infection, haematoma, ptosis, wound dehiscence) in evisceration and enucleation cohorts. n=73 (evisceration=45, enucleation=28). A p-value <0.05 was considered statistically significant between the evisceration and enucleation cohorts.

The postoperative complications experienced collectively among the 73 eyes were implant exposure or extrusion (n=6, 8.2%), inflammation or infection (n=4, 5.5%), wound dehiscence (n=3, 4.1%), hematoma (n=2, 2.7%), and ptosis (n=2, 2.7%). There was no reported SO among the two cohorts. There was no statistical difference between the prevalence of postoperative hematoma, infection, wound dehiscence, or ptosis between the evisceration and enucleation cohorts. There was, however, a higher prevalence of implant complications in the enucleation cohort in comparison to the evisceration cohort (p=0.029) (Figure 1).

Postoperative complications according to participant and implant characteristics

Patients who had postoperative issues following evisceration were all Caucasian patients, while patients who had postoperative issues following enucleation were a mixture of Caucasian patients (n=8, 80.0%) and Aboriginal and Torres Strait Islander or Other individuals (n=2, 20.0%) (p=0.004). Patients who had undergone evisceration due to non-healing corneal perforation or painful blind eye had a greater prevalence of postoperative complications, whereas patients who had undergone enucleation surgery had postoperative complications regardless of the indication for surgery (p=0.022). The prevalence of implant complications between evisceration and enucleation was not statistically significant for birth sex, race, surgery indication, implant material, or implant size (Table 4).

**Table 4:** Postoperative issues and implant extrusion depending on sex, ethnicity, surgery indication, implant material, and implant size between evisceration and enucleation cohorts.

		Postoperative complication (n=17)			Implant exposure or extrusion (n=6)		
Variable		Evisceration (n=7)	Enucleation (n=10)	p-value	Evisceration (n=1)	Enucleation (n=5)	p-value
Sex	Female	3 (42.8%)	5 (50.0%)	0.201	1 (100.0%)	2 (40.0%)	0.273
	Male	4 (57.2%)	5 (50.0%)		0 (0.0%)	3 (60.0%)	
Race	Caucasian	7 (100.0%)	8 (80.0%)	0.004	1 (100.0%)	4 (80.0%)	0.624
	Aboriginal and Torres Strait Islander or Other	0 (0.0%)	2 (20.0%)		0 (0.0%)	1 (20.0%)	
Surgery indication	Non-healing corneal perforation	3 (42.9%)	1 (10.0%)	0.022	1 (100.0%)	0 (0.0%)	0.112
	Malignancy	0 (0.0%)	1 (10.0%)		0 (0.0%)	0 (0.0%)	
	Painful blind eye	4 (57.1%)	3 (30.0%)		0 (0.0%)	1 (20.0%)	
	Phthisical eye	0 (0.0%)	0 (%)		0 (0.0%)	1 (20.0%)	
	Trauma	0 (0.0%)	5 (50.0%)		0 (0.0%)	3 (60.0%)	
Implant material	Acrylic	7 (100.0%)	9 (90.0%)	0.709	1 (100.0%)	5 (100.0%)	0.264
	Glass	0 (0.0%)	0 (0.0%)		0 (0.0%)	0 (0.0%)	
	Porous polyethylene	0 (0.0%)	1 (10.0%)		0 (0.0%)	0 (0.0%)	
Implant size	16mm	1 (14.3%)	0 (%)	0.505	0 (0.0%)	1 (20.0%)	0.157
	18mm	0 (0.0%)	0 (%)		0 (0.0%)	0 (0.0%)	
	20mm	5 (71.4%)	5 (50.0%)		1 (100.0%)	2 (40.0%)	
	22mm	1 (14.3%)	5 (50.0%)		0 (0.0%)	2 (40.0%)	

## Discussion

In this study, demographic and surgical characteristics, along with postoperative outcomes, were compared between evisceration and enucleation cohorts over a 10-year period at a major Australian tertiary public hospital. More than one-third of patients who underwent enucleation surgery experienced postoperative complications and had a significantly higher prevalence of implant-related issues, such as exposure or extrusion, compared to those in the evisceration cohort. Patients who underwent evisceration for a painful blind eye or non-healing corneal perforation were more likely to develop postoperative complications than those who had the procedure for other indications. Similarly, enucleation performed due to trauma accounted for the majority of postoperative complications within this cohort. Despite these findings, no cases of SO were reported in either the evisceration or enucleation groups, challenging the assumption that evisceration carries a higher risk of SO than enucleation. By the one-year postoperative mark, nearly half of the evisceration cohort and one-quarter of the enucleation cohort were lost to follow-up. This highlights the need for nationwide Australian guidelines to establish standardized surgical guidelines and recommended follow-up durations for anophthalmic surgery.

SO is a rare, non-infectious, bilateral granulomatous panuveitis that can occur following ocular trauma in one eye due to a mediated immune response [3,11-14]. A 36-year review by Bui et al. reported an SO incidence of 0.2% (20 out of 9,092 eyes) in patients who had undergone enucleation or evisceration, with no significant difference between surgical and non-surgical trauma as an SO etiology [3]. In contrast, Mansouri et al. described a lower SO incidence of 0.08% in 2,340 eyes; however, this study covered only a five-year timeframe [15]. Despite these low incidences, traditional guidelines recommend enucleation within fourteen days of an ocular injury to protect the contralateral eye from SO [16]. In our study, four cases of postoperative inflammation or infection were identified collectively, though none were due to SO. One patient developed a potentially early panuveitis on day 46 post-evisceration. However, this was attributed to acute retinal necrosis secondary to a positive HSV-1 result from the eviscerated specimen and was not classified as SO. This patient had a negative fundus fluorescein angiogram and responded well to antiviral and steroid therapy. Freidlin et al. previously reported a case of SO occurring three weeks after evisceration, which was likely due to residual uveal tissue in the orbit, discovered during re-exploration after wound dehiscence [12]. A 2022 systematic review by Jordan and Dutton estimated the risk of SO development from all causes with predisposing factors (e.g., globe trauma, intraocular foreign body, vitrectomy) to be extremely low: one in 100,000 (0.001%) for enucleation and one in 55,000 (0.002%) for evisceration. Their findings support the conclusion that evisceration is an acceptable surgical choice with minimal risk of SO [17]. Similarly, Ullrich et al. reported comparable SO risks post-enucleation (one in 1,700,000) and post-evisceration (one in 840,000), likening these to the risk of death from general anesthesia for elective surgery (one in 100,000) [18]. Our ten-year retrospective chart review aligns with these findings, as no cases of SO were reported in either the evisceration or enucleation groups. The role of enucleation as a prophylactic measure has become increasingly controversial, as recent literature suggests that it does not entirely eliminate the risk of SO [11,13,14]. Tseng et al. described a case of SO following enucleation, noting that factors such as previous vitrectomy and retinal detachment may have contributed to SO development [14]. Given our findings and current literature, the assumption that enucleation is the safer option for SO prevention should be reconsidered. Enucleation may only be necessary in cases involving uveal spread beyond the sclera or intraocular malignancy. Further research incorporating confounding variables in a multisite, longitudinal setting is recommended.

In our study, evisceration performed for a painful blind eye or non-healing corneal perforation was more likely to result in postoperative complications in comparison to other surgical indications. In contrast, enucleation due to trauma accounted for most postoperative complications in this cohort, which may relate to the distortion or loss of normal orbital tissues. While the prevalence of wound dehiscence was similar between the evisceration and enucleation groups, there was a significant difference in implant complication rates. Further research into the risk of developing implant exposure or extrusion from wound dehiscence should be explored. For all patients, almost 6% (n=4) experienced postoperative inflammation or infection, with no statistical difference between evisceration and enucleation. There was, however, a significant difference in postoperative complications, with 35.7% (n=10) of patients in the enucleation cohort experiencing a postoperative complication in comparison to 15.6% (n=7) in the evisceration cohort.

Table 5 summarizes English-language studies from the past two decades that analyze and compare postoperative complications, implant exposure, and/or SO rates between primary evisceration and enucleation surgeries. A 2006 study by Nakra et al. found no differences in aesthetic outcomes between enucleated and eviscerated eyes [6]. However, evisceration resulted in significantly better implant motility and fewer postoperative complications [6]. The study reported a postoperative complication rate of 21.9% for enucleation and 13.5% for evisceration [6]. In contrast, a 2012 study by Yousuf et al., which spanned 20 years, found no significant difference in postoperative complications between the two procedures [19]. However, evisceration required significantly less surgical time, thereby reducing surgical stress on the patient [19]. Similarly, a retrospective chart review by Zheng et al. in 2012, analyzing 21 eyes at a New York trauma center, reported no cases of SO following either procedure [20]. The study highlighted that surgical decision-making was largely dependent on surgeon preference and experience [20].

**Table 5:** Characteristics of past studies analysing primary evisceration and enucleation surgery and associated rates of postoperative complications and/or sympathetic ophthalmia (SO).

Author (publication year)	Country	Study design	Timeframe of review (years)	Primary surgery	No. of eyes	Postoperative complication rate (%)	Implant exposure rate (%)	SO rate (%)
Nakra et al. (2006) [6]	USA	Retrospective review	5	Evisceration	52	13.5	3.8	N/A
				Enucleation	32	21.9	12.5	
Yousuf et al. (2011) [19]	USA	Retrospective review	20	Evisceration	54	55.6	11.0	0.0
				Enucleation	31	83.4	16.0	
Zheng et al. (2012) [20]	USA	Retrospective review	11	Evisceration	6	16.7	0.0	0.0
				Enucleation	16	12.5	0.0	
McElnea et al. (2013) [21]	Ireland	Retrospective review	5	Evisceration	14	-	37.5	N/A
				Enucleation	24	-	62.5	
Valeshabad et al. (2014) [22]	Iran	Retrospective review	5	Evisceration	7	44.8	12.5	N/A
				Enucleation	100			
Rebollo et al. (2022) [23]	Puerto Rica	Retrospective review	5	Evisceration	22	9.1	-	N/A
				Enucleation	85	9.4	-	
Zhang et al. (2015) [24]	China	Retrospective review	20	Evisceration	406	-	8.5	0.0
				Enucleation	167	-	10.0	
Ababneh et al. (2015) [1]	Jordan	Retrospective review	5	Evisceration	42	71.4	7.1	0.0
				Enucleation	26	61.5	11.5	
Al-Farsi et al. (2017) [8]	Oman	Retrospective review	6	Evisceration	26	-	0.0	N/A
				Enucleation	11	-	0.0	
Svedberg (2023) [5]	Sweden	Retrospective review	10	Evisceration	166	20.5	0.0	N/A
				Enucleation	84	14.3	3.6	

Following these early studies, researchers explored other confounding factors affecting postoperative outcomes. A retrospective chart review in the United Kingdom involving 38 eyes found significantly lower implant exposure rates when enucleation or evisceration was performed by an orbital surgeon (4%) compared to non-orbital surgeons (48%), underscoring the importance of surgical expertise in postoperative outcomes [21]. A 2014 review from a tertiary hospital in Iran found that enucleation was associated with a higher incidence of implant-related and postoperative complications when hydroxyapatite implants were used. This was possibly influenced by the subtropical climate and patient challenges with postoperative wound care [22]. More recently, a retrospective review published in 2023 from a Swedish hospital reported a significant reduction in implant-related complications following modifications in surgical technique and the use of smaller implants in evisceration surgeries [5]. These findings suggest that orbital surgical expertise, perioperative management, and patient education may play crucial roles in postoperative outcomes following anophthalmic surgery and should be investigated further.

Patient demographics and social environments also influence surgical decision-making. A 2022 retrospective study from a supratertiary Puerto Rican hospital found a significantly higher number of enucleation surgeries compared to evisceration, largely due to a high prevalence of trauma cases and the influence of patient risk factors and surgeon preference [23]. Similarly, a 20-year retrospective study of 573 eyes in China reported lower implant exposure rates following evisceration compared to enucleation, with no reported cases of SO [24]. In developing countries, trauma remains the primary clinical indication for enucleation [24]. Although our study reported fewer trauma cases compared to those in developing nations, promoting ocular safety education is crucial in reducing the incidence of severe ocular injuries and subsequent enucleation, emphasizing the importance of primary prevention.

In our study, the incidence of implant complications did not show statistically significant differences based on birth sex, race, surgical indication, implant material, or implant size between the evisceration and enucleation cohorts. One patient in the evisceration cohort experienced implant extrusion, while five patients in the enucleation cohort developed implant exposure, three of whom had undergone enucleation due to trauma. However, no statistically significant association was found between surgical indication and the prevalence of implant complications across the groups. These findings align with a case-control study by Gupta et al., which identified no definitive risk factors for implant exposure [25]. However, their study observed an increased risk of implant exposure with porous implant material, prior ocular surgery, and infection, although these were non-significant trends [25]. Similarly, Kim et al. reported that implant size and hydroxyapatite implants were significant risk factors for implant extrusion in patients who underwent evisceration [26]. Although our study found a higher occurrence of implant exposure in 20 mm and 22mm orbital implants, this difference was not statistically significant, nor was there a significant difference between the evisceration and enucleation cohorts. Other confounding variables, such as surgical technique and postoperative care, may influence clinical outcomes and should be explored further, particularly within the Australian landscape.

Orbital implants are used to restore socket volume and contour following enucleation or evisceration and can be made from various materials [8,9,27,28]. Non-porous orbital implants (e.g., acrylic) are cost-effective but have reduced motility and are believed to carry a higher risk of migration compared to porous implants [27-29]. In contrast, porous orbital implants (e.g., porous polyethylene) allow fibrovascular ingrowth, which theoretically enhances implant retention within the orbit [27,29]. Wladis et al. reported that the incidence of implant extrusion for non-porous implants ranged from 0% to 7.1%, while extrusion rates for porous implants ranged from 0% to 1.3% [28]. However, they noted that the higher extrusion rates observed for non-porous implants may be attributed to longer follow-up durations in studies that included them [28]. In our study, six patients (8%) experienced implant complications, all of whom had acrylic orbital implants. However, given that approximately 90% of the implants used in our study were acrylic, this may not accurately reflect the true impact of implant material on the incidence of exposure or extrusion. Similarly, Ho et al. retrospectively analyzed 416 patients who had undergone primary enucleation for uveal melanoma and found no significant differences in complication rates or patient satisfaction between porous and non-porous implants [27]. Conversely, a 21-year review of enucleation and evisceration cases with porous implants by Lin et al. reported implant exposure rates of 24.7% for hydroxyapatite, 23.5% for bioceramic, and 76.5% for porous polyethylene [30]. These rates were significantly higher than those reported in other studies, though their follow-up period was notably longer, with the average time to implant exposure being 67.4 months for hydroxyapatite, 52.5 months for bioceramic, and 73.4 months for porous polyethylene [30]. These findings suggest that postoperative implant complications following anophthalmic surgery may be more frequent than previously described. Additionally, past literature has been limited by small sample sizes, inconsistent follow-up, and confounding variables such as surgical technique, surgeon experience, and the wide variety of available implants [29]. In our study, one-quarter (n=7) of the enucleation cohort and nearly half (n=22) of the evisceration cohort were lost to follow-up by the one-year postoperative mark. This highlights the need for improved patient education on the importance of postoperative follow-up, particularly given that implant exposure can remain a risk years after surgery. Further investigation into qualitative factors (e.g., education level, occupation) that may influence compliance with follow-up appointments is warranted. Identifying these risk factors could help mitigate postoperative complications and implant exposure risk.

Strengths and limitations

The strengths of this study include longitudinal data collected from a major tertiary Australian hospital, which is the largest in the state. It still remains a single-site study; therefore, these results may not provide complete generalizability to other developed and/or developing nations. Given its retrospective nature, the accuracy of this data is reliant on documentation completeness and identification of evisceration/enucleation cases based upon procedural hospital coding. Many patients were lost to follow-up in both cohorts; therefore, the number of postoperative complications or need for subsequent implant revision may be underestimated. Irrespective of this, given that this data collection was obtained from the largest tertiary adult ophthalmology center in the state, it may be reasonable to assume that if patients did have a postoperative complication, they would have reported to the facility. Other variables, such as surgical technique, may have also contributed to the results, which should be explored in future studies. Many of the enucleation cases due to malignancy were followed up and managed by the Queensland Ocular Oncology Service and were not included in this study due to a lack of baseline data; therefore, postoperative details and outcomes may also be underestimated in this subgroup of patients. Future studies should also include multi-centers to compare and contrast the differences between varying health services. This information may therefore highlight the need for a standardized guideline in Australia.

## Conclusions

There were no documented cases of SO in either the evisceration or enucleation cohorts. However, patients who underwent enucleation surgery were significantly more at risk of experiencing postoperative complications, such as implant exposure and implant extrusion. There is poor follow-up compliance in patients who have undergone anophthalmic surgery in the Australian public hospital system. Improving patient education and creating nationwide Australian surgical guidelines may help to achieve better postoperative outcomes and follow-up durations after anophthalmic surgery. Future studies should analyze other confounding variables such as surgical technique and patient perspectives, alongside including multicenter data to address literature gaps.
